# Radiographic ratios for classifying furcation anatomy: proposal of a new evaluation method and an intra-rater and inter-rater operator reliability study

**DOI:** 10.1007/s00784-022-04774-6

**Published:** 2023-02-13

**Authors:** Enrico Limiroli, Andrea Calò, Alessandro Limiroli, Ivan Cortinovis, Giulio Rasperini

**Affiliations:** 1 Department of Biomedical, Surgical and Dental Sciences, University of Milan, Via Della Commenda 10, 20122 Milan, Italy; 2 Foundation IRCCS Ca’ Granda Polyclinic, Via Della Commenda 10, 20122 Milan, Italy; 3 Private Practice, Milan, Italy; 4 Private Practice, Bergamo, Italy; 5 Laboratory G.A. Maccacaro, Department of Clinical Sciences and Community Health, University of Milan, Milan, Italy

**Keywords:** Furcation anatomy, Furcation measurement, Furcation evaluation

## Abstract

**Objectives:**

Even if it seems to be an important anatomical parameter for tissue regeneration, few studies in literature evaluate the “mean measure” of root divergence. Most of them are linear measurements, which hardly describe the dental furcation conformation in its entirety. It is left to the subjectivity of the operator deciding whether a furcation is convergent or divergent. The goal of this study is to create a visual evaluation method using specific measurements applied on endo-oral X-rays to overcome these problems, giving a conformation of the entire interradicular space and its divergence.

**Material and methods:**

A user-friendly software (Paint®, Windows10®) was used to take three different measurements on endo-oral radiographs of upper and lower molars. Three blind operators measured 20 radiographs, to analyze the intra- and inter-operator reproducibility of the measurements. Then, the technique was repeated on 250 radiographic images to identify an average value and define a main conformation of the interradicular space. The ratio of these three measurements allowed to develop a new visual evaluation method of the interradicular space.

**Results:**

Intra and inter-operator reproducibility was statistically confirmed on a sample of 20 anonymous endo-oral radiographs measured by 3 blind operators, indicating that the measurement technique was not operator dependent. Measurement made on 250 X-rays obtained with this technique permitted to subdivide in five groups the conformation of the interradicular space and define a mean value of the interradicular space.

**Conclusions:**

A new anatomical evaluation of the interradicular space in its entirety, which could help the clinicians in diagnostic and decisional phase in the therapy of furcated molars, can be obtained.

**Clinical relevance:**

A pre-operative evaluation of interradicular space conformation could affect therapy treatment choice.

## Introduction

Molar teeth with furcation defects are considered one of the main challenges in periodontal therapy. While Class I furcations are generally maintainable with non-surgical therapy alone [1], Class II furcations can be successfully treated with regenerative approaches, leading to a reduction in horizontal and vertical probing and a clinical attachment gain [2–7]. From a biological point of view, the regenerative potential within the furcation area is limited, and it seems to be conditioned by several anatomical factors that may increase the difficulty of the regenerative procedure. The proportion of keratinized gingiva on the affected tooth, the phenotype of the patient [8, 9], and the severity of the bone defect, both horizontal (degree I, II, III) [10] and vertical (A, B, C according to Tarnow, 1984 [11] and subclasses A, B, C Tonetti 2007 [12]) are all influencing factors. Finally, the root anatomy (both the divergence of the furcation and the length of the root trunk) has been repeatedly demonstrated influential [13–16], although some authors do not consider it a success/failure factor [17]. Nevertheless, in literature there are only few studies evaluating the “mean measure” of root divergence, or the amplitude of the inter-root septum. In their study, Bowers et al. measured the divergence as root divergence at crest of the bone (RDCB) [14]. Caesarin et al. [18] took a linear measurement after flap elevation and bone defect debridement. In this case the authors meant “divergence” the distance between the roots 2 mm under the fornix of the furcation. With these measures, it is difficult to describe the divergence coronally or apically the measuring site. For example, if a tooth has convergent root cones in its apical portion, this will not be considered. Moreover, these are intraoperative clinical measures; therefore, they do not allow to establish before the surgery the divergence of the furcation and again these are single measures. It is therefore left to the subjectivity of the operator to decide whether a fork is convergent or divergent. The goal of this study is to create a visual evaluation method that can overcome these problems giving a conformation of the entire area of the interradicular space. Therefore, in this study the root conformation is described by the width of the roof of the furcation and the width of the space between the two roots [14–20]. The larger the width of the furcation roof, the easier the debridement of the area will be, but at the same time, it will be more difficult obtaining a complete closure of the defect due to an inability of the clot to effectively fill the area. For the same reason, increased root divergence is associated with a larger furcation defect, which may result in reduced horizontal bone gain, reduced furcation closure, and unfavorable regenerative outcome [19]. Putting together these two parameters, transversal width and furcation roof related to the interradicular septum length, gives us two ratios which describe the anatomy of the interradicular space in its entirety. Also, since the measurements are ratios, they are not subjected to image scale errors of measurements. This method is applicable only on clear and non-overlapped X-rays of lower molars and on the vestibular furcation of upper molars, since it is impossible to obtain clear and non-overlapped X-rays on the upper palatal side.

Therefore, the aim of the present study is to propose a method for describing roots conformation and to test its intra- and inter-rater reliability.

## Materials and methods

### Study design

This is an intra-rater repeatability and inter-rater test–retest reliability study. Three blind operators measured 20 intraoral radiographs of upper and lower molars to evaluate the reproducibility of the measurement technique. A pre-calibrated operator measured 250 intraoral radiographs of lower molars for the definition of medium radicular space in molars vestibular furcation. The X-rays were collected using the DBSWIN software (Version 5.17.0, DÜRR DENTAL SE, Germany) in a progressive numbering archive.

### Inclusion criteria

Periapical radiographs of upper and lower molars were retrieved. On upper molars, only the vestibular furcation was evaluated.

Radiographs were included in the present study if they met the following inclusion criteria:

- Radiographs made with paralleling technique
- Good image definition (clearly distinguishable roots margins)
- Complete view of the interradicular space
- Complete view of root apices

### Exclusion criteria

Radiographs with extreme roots overlapping, incomplete view of interradicular space, or any other reason which made the images unreadable were excluded.

### Novel method for classifying the furcation divergence in mandibular molars

The novel method is based on the assessment of the following parameters on periapical X-rays:

- Furcation roof width (*D*)
- Interradicular furcation width (*T*)
- Interradicular vertical septum length (*L*)

**Fig. 1:**
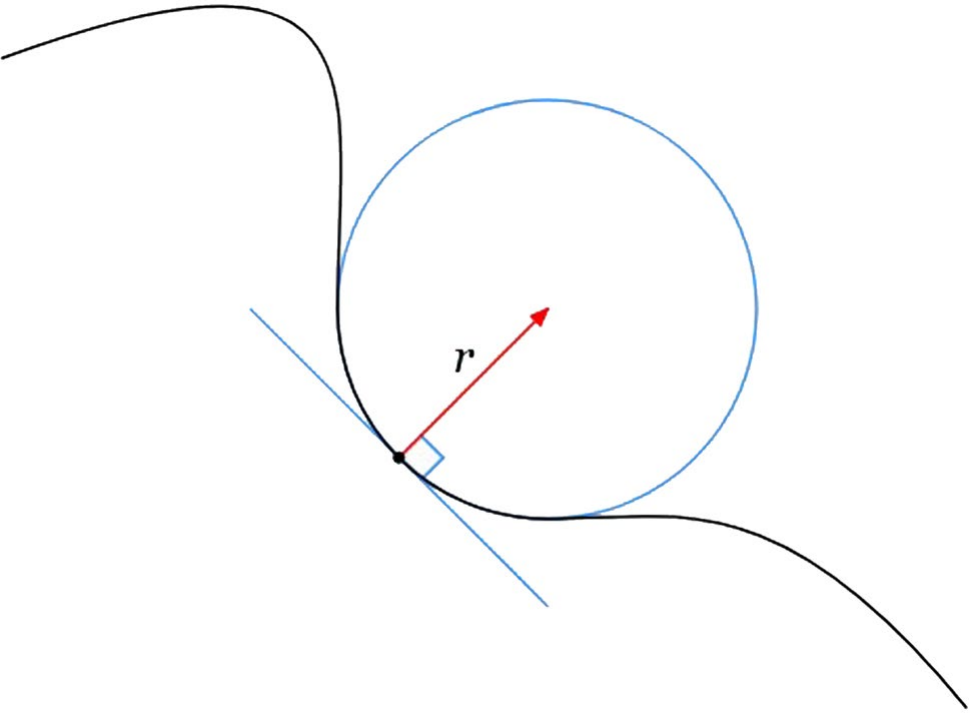
Example of an osculating circle: Just as the tangent line is the line best approximating a curve at a point, the osculating circle is the best circle that approximates the curve at

Intraoral radiographs made with paralleling technique of upper and lower molars studied. In upper molar only the vestibular furcation was considered. Using a graphic software (Paint®, Windows10®), a vertical line was drawn from the roof of the furcation to the midpoint of an imaginary line passing through both root tips. This line reflected the vertical length of the interradicular septum. A second horizontal line was drawn within two thirds coronal of the interradicular septum, where the interradicular septum had the maximum width. Lastly, an “osculating circle” was traced along the curvature of the furcation roof to extrapolate the width of the roof itself. The osculating circle (Fig. 1) is a geometrical concept: it is the best circle that approximates a curve at. Tracing a circle overlapping as much as possible the furcation roof curvature, lets us define with a geometric precision the curvature of the given furcation. In case of a furcation roof with two overlapping curvatures, possibly due to superimposition on the X-ray, the mean curvature was selected.

**Fig. 2:**
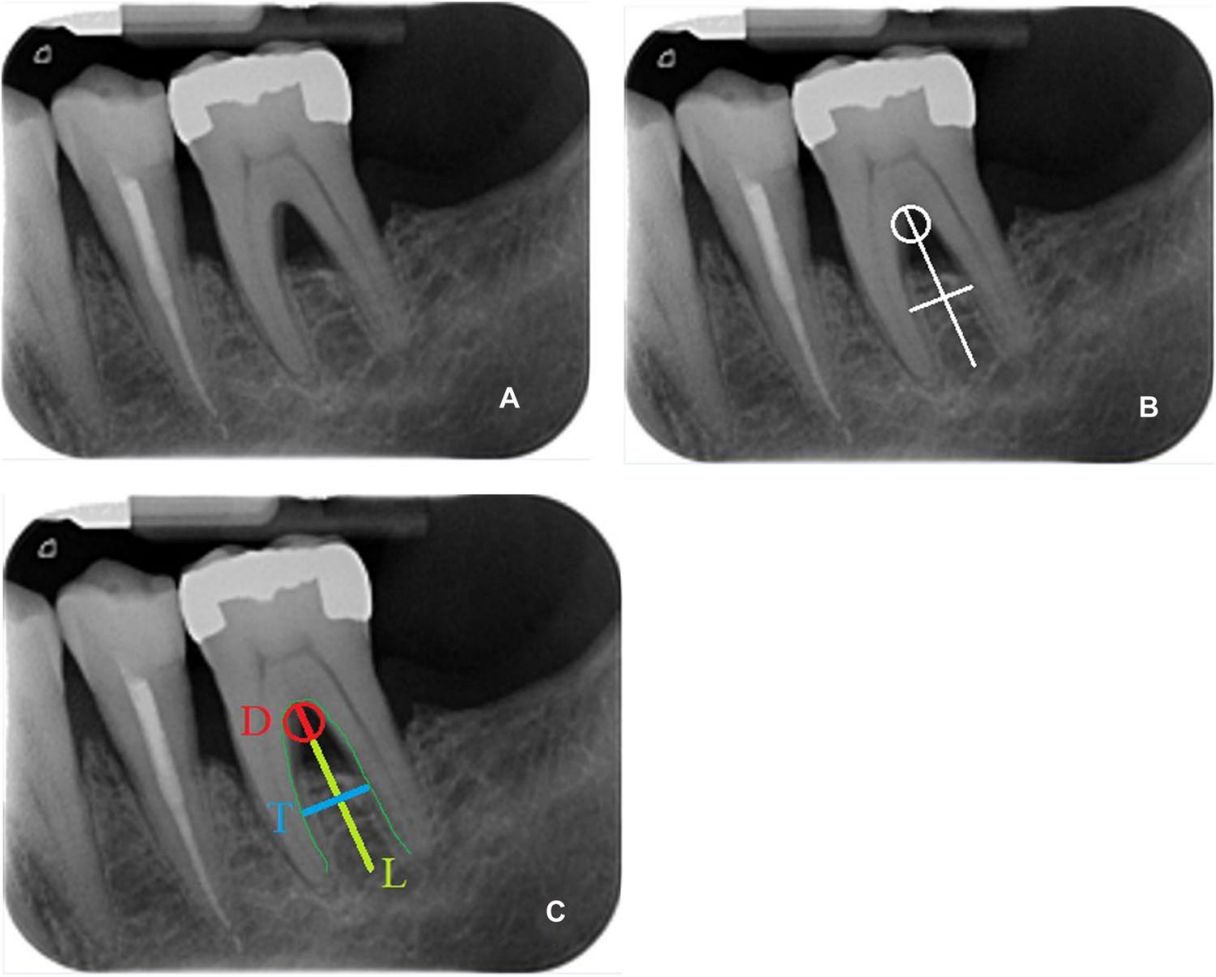
**A** A peri-apical X-ray lower molar. **B** The visual evaluation method. The osculating circle, the trasversal width and the longitudinal length are drawn on the rx. **C** Diameter of the osculating circle (*D*, in red); trasversal width (*T*, in light blue); longitudinal length (*L*, in yellow)

The diameter and the lines are measured on the same graphic program (Paint®, Windows10®). Ratios are created as follows: between the diameter of the circle (*D*) and the length of the line from the roof of the furcation to the midpoint of an imaginary line passing through the tips of the roots (*L*) and between the line drawn in the maximum width of the interradicular septum (*T*) and the line from the roof of the furcation to an imaginary line passing through the tips of the roots (*L*) (Fig. 2).

The *D*/*L* ratio reflects please consider replacing “express” with “reflects” the roof furcation width (RFW), and the *T*/*L* reflects same as before the interradicular furcation width (IFW).

### Intra-rater and inter-rater operator reliability assessment of the new method for characterizing furcation divergence

Three dentists (E.L., A.L., P.B.), without previous knowledge of the selected cases, blindly assessed furcation divergence with the proposed evaluation system on a sample of 20 periapical X-rays. The 3 operators carried out the measurements of *D*, *T*, and *L* and calculated their ratios at time 0 (T0). After 30 days (T1), the operators repeated the same evaluation on the same 20 cases. All the measurements were made independently each operator from the other.

### Determination of the “mean furcation conformation/configuration”

A sample of 249 periapical radiographs was collected by a pre-calibrated operator (A.C.), who measured *D*, *T*, and *L* and the ratios *D*/*L* (RFW) and *T*/*L* (IFW). The ratios were then statistically analyzed and an average measure of the reports, reflecting the interradicular space, was created, based on the statistical distribution of the measurements collected.

### Statistical analysis

The reliability of the novel evaluation of furcation divergence was explored among the same examiner at different timepoints (intra-reliability) and between different examiners (inter-reliability) on 20 periapical X-rays. Bland–Altman plots were constructed to investigate the intra-rater and inter-rater operator reliability. A generalized linear model was used to evaluate the repeatability of the two measurements in relation to the operators (fixed effects) and the measurements carried on the same objects (random effects).

## Results

### Intra-rater and inter-rater operator reliability assessment

The linear generalized model for RFW as dependent variable showed a variability (*R*-square) of 83.4% and that the investigator seems to have a significant role in determining the measurements (*p* < 0.01). Examiner 1 was found to give statistically significantly different measurements compared to operator 2; however, the difference between the two operators, and overall, among each operator, are < 5%.

The linear generalized model for IFW as dependent variable showed a variability (*R*-square) of 94.8%, with the operator having a significant role in the model (*p* < 0.05). A statistically significant difference between examiner 1 and 3 was observed (*p* < 0.05); however, this difference was within 5%.

**Fig. 3:**
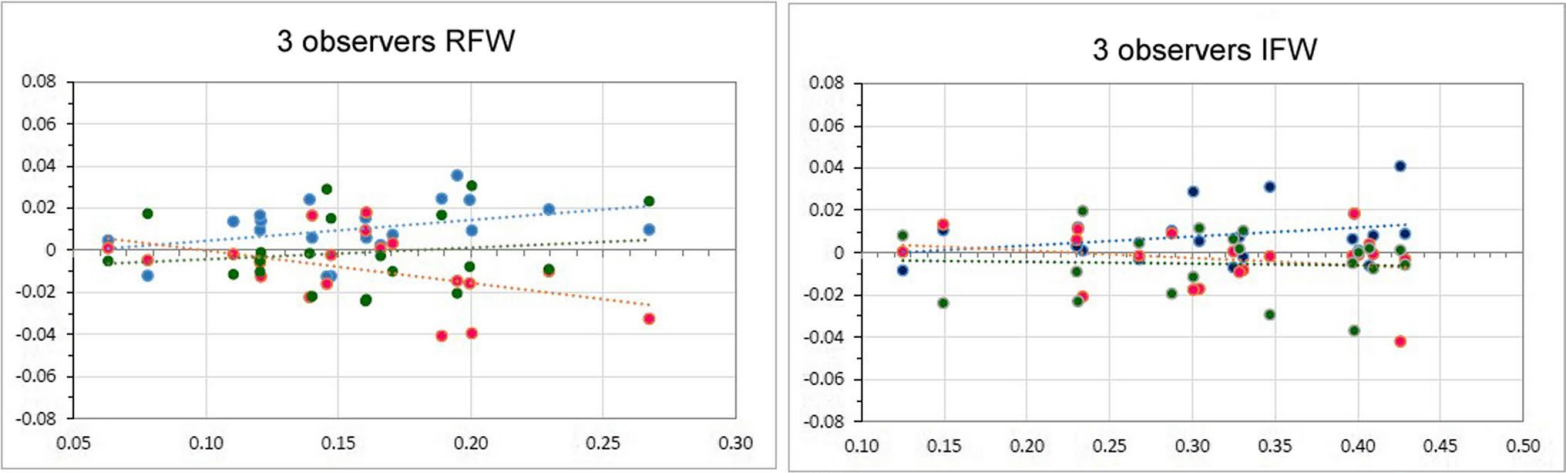
Graphs from the linear generalized model using the RFW ratio (**A**) and the IFW ratio (**B**) as dependent variables, showing the mean differences in measuring these ratios obtained by the operators. The graphs show negligible differences among the examiners, less than 5%, confirming that the described method is reproducible and consistent

Overall, the two linear generalized models showed that are no differences between the two sets of measurements performed by the same operators at T0 and T1 (*p* > 0.05). Although a statistically significant different was found between the measurements of two examiners for RFW and IFW, these differences were within 4 hundredths of a millimeter (< 5% of the original measurements) and can therefore be considered not clinically relevant and negligible (Fig. 3).

### Determination of the “mean furcation conformation/configuration”

Based on 249 periapical X-rays, the mean furcation conformation/configuration was characterized in terms of RFW and IFW. The average RFW and IFW were 0.163 and 0.307, respectively. Table 1 shows the quintile division of the obtained distribution.

**Table 1:** Distribution of subjects for every quintile for RFW and IFW

Quintiles RFW	*N*
1 (0.01–0.07)	11
2 (0.07–0.15)	110
3 (0.15–0.22)	96
4 (0.22–0.30)	26
5 (0.30–0.38)	4
Quintiles IFW	*N*
1 (0.06–0.14)	20
2 (0.14–0.29)	102
3 (0.29–0.43)	95
4 (0.43–0.58)	29
5 (0.58–0.73)	4

## Discussion

It has been well established that the anatomy of the roots, both in terms of furcation divergence and length of the root trunk, is associated with outcomes of periodontal regeneration [13–20]. Nevertheless, only few studies have focused on measuring parameters related to anatomy of the roots/furcation, including fork divergence, root trunk length, radicular roof width, and interradicular width [14–20]. Previous evaluation systems introduced in the past for describing furcation involvement often lack reliability assessment.

The aim of the present study was to propose a visual evaluation method of assessing furcation/root anatomy and, in line with the goals of contemporary literature, to test the intra-rater and inter-rater reliability of the proposed system. We described an objective method of identification and measurement of the interradicular space in two dimensions. Results from the reliability study demonstrated that this method is reliable and consistent among the same operator at different time point and among different examiners. Therefore, this novel method can be easily performed by all clinicians. Furthermore, the measurements on radiographic images can be obtained by using basic and “user-friendly” graphic software compatible with all kinds of hardware.

To the best of the authors’ knowledge, a similar evaluation characterizing the entire radicular space has not been described in the literature so far. This may have a positive impact on future investigations exploring the impact of RFW and IFW on the treatment outcomes of periodontal regeneration.

There are individual parameters such as divergence, degree of separation, and separation coefficient that however failed to provide an overview of the radicular space, when taken individually investigated. For example, the method proposed by Bowers et al. to measure the divergence as root divergence at crest of the bone (RDCB) [14] does not provide a pre-operative way to analyze the root divergence. Another method of evaluation of the divergence was proposed in the study of Caesarin et al. [18], who, after flap elevation and bone defect debridement, calculated the distance between the roots 2 mm under the roof of the furcation. These linear measures do not provide complete information on the entire root space. There are, indeed, several cases in which furcated molars have a large roof and converging roots, or a narrow roof and diverging roots, where it is difficult to determine whether the interradicular space is large or narrow. The predictability in the regenerative therapy could be obtained with pre-operative measures. In this study the measurements are ratios between lines drawn by a basic graphic software. The advantage of the proposed evaluation is the fact that the common problem of image magnification on X-rays are overcome by the calculation of ratios, that are independent from the scale of measurement and do not suffer of scale errors. Magnifications caused by the position of the sensor relative to the tooth and by the manipulation of the digital image by the operator do not affect the accuracy of the proposed evaluation. When these lines are examined together, they give the operator the shape of the interradicular space. The proposed evaluation allows to have a global vision of the interradicular space. Also, since the previous divergence measurement techniques were only intraoperative, they cannot be used as a diagnostic method. The method herein described, besides being more complete in the description of the interradicular space, it is feasible in diagnostic phase. This could be helpful in clinical treatment choice.

Subdivision in quintiles of the range of measurements of RFW and IFW (Table 1), showed a higher percentage of population, for both ratios, in the second and third quintiles. This means that a more frequent furcation conformation could be extrapolated. Analyzing the conformation of molars which received regenerative treatment and combining the different groups of distribution in quintiles with regenerative success could confirm the influence of divergence in tissue regeneration and which conformation holds much regenerative potential.

Among the limitations of the present study, the lack of standardization of the periapical X-rays utilized to conduct the reliability study and the determination of the “mean furcation configuration” must be mentioned. Also, it is not possible to apply this method to the palatal furcation of upper molars due to overlapping of the mesial and distal roots. In addition, the sample size is relatively limited and represent a specific population only and therefore further studies are needed to explore the generalizability of this method in other populations. Lastly, although this preliminary study provides the basis for utilizing this evaluation system in everyday case scenarios, clinical studies are needed to evaluate whether the root/furcation anatomy, described with the proposed method and ratios, affects the outcomes of periodontal therapies.

## Conclusions

The present study introduced a new anatomical evaluation of the interradicular space, which could help clinicians and researchers when searching for an objective quantitative description of the furcation configuration/anatomy. This visual evaluation method has proven to be reliable and consistent among the same operators and different examiners. Subdivision in quintiles of the range of measurements could establish a classification of the interradicular area based on the value of more representative interradicular space. The ability to measure these values and to classify them objectively may allow to weigh their influence on bone regeneration and improve the way bone defects are managed. Further studies are needed to establish a possible positive correlation with the regenerative potential.
